# Data altruism and the “consent” question: a study into the “consent” models used under the GDPR and how the data altruism mechanism can act as a potential solution for the research community in the reuse of health data

**DOI:** 10.3389/fmed.2024.1489925

**Published:** 2025-02-25

**Authors:** Maria Christofidou, Theodoros N. Arvanitis, Dipak Kalra, Nathan Lea, Mahsa Shabani, Pascal Coorevits

**Affiliations:** ^1^Department of Public Health and Primary Care, Faculty of Medicine and Health Sciences, Ghent University, Ghent, Belgium; ^2^Department of Electronic, Electrical and Systems Engineering, University of Birmingham, Birmingham, United Kingdom; ^3^The European Institute for Innovation through Health Data, Ghent, Belgium; ^4^Faculty of Law and Criminology, Ghent University, Ghent, Belgium; ^5^Law Centre for Health and Life, Amsterdam Law School, University of Amsterdam, Amsterdam, Netherlands

**Keywords:** GDPR, consent, data altruism, data governance act, health data, secondary use of data, data sharing

## Abstract

**Introduction:**

The General Data Protection Regulation (“GDPR”) legal basis for obtaining consent for the processing of personal data for research purposes, where those purposes cannot be fully specified in advance, is provided for in Articles 6, 7 and Recital 33. However, GDPR’s requirements for obtaining consent, as to the secondary use and sharing of data in research, have been argued to have generated confusion, whilst the conflicts between the Regulation itself, its practical application and research ethics are well-documented (1). The requirements for “informed consent”, as defined within the GDPR, have not been well defined in the context of genome research or clinical trials (2), which has in turn led to the implementation and interpretation of the lawful basis to span into different idiosyncratic models. This naturally has fed into the uncertainty of how the legal basis can be applied in practice and calls for an investigation into the requirements for consent to be “informed” in the context of health research. This work aims to provide a scoping review and analysis of relevant publications with ultimate purpose to examine whether the concept of ‘data altruism’, as stipulated within Article 2 (10) of the Data Governance Act (“DGA”), addresses the gaps left behind by the application of the legal basis of ‘consent’, under the GDPR (Art. 6 (1) and 7), in so far as the secondary uses of data for research are concerned. In this light the article, by exploring available solutions found in relevant literature and used in practice in national and European projects, examines how ‘data altruism’ can add any value and work as a cohesive solution that the research community can use.

**Objectives:**

The article, through its research, intends to answer the following questions:

1. What gaps has the GDPR left when it comes to the interpretation and practical application of “consent” towards the secondary use of health data;
2. Can the DGA, through the mechanism of ‘data altruism’, address these issues and provide a solution;
3. What solutions have been used so far in practice to address this issue.

**Methodology:**

To address the above-mentioned questions, the Arskey and O’Malley scoping review methodology and best practice, as outlined in the Joanna Briggs scoping review guidelines, have been applied. The research questions have been identified through an extensive literature review and consultation with subject matter experts. The search was conducted using six search engines and utilising a tailored search strategy, with the application of both MESH and non-MESH based search terms. From the identified relevant publications, 148 abstracts were kept to be read and 60 of those publications were kept as relevant. A PRISMA chart showcases the process in which the publications were reviewed and the process which led to the final papers kept as relevant. The title-abstract and full text screening and charting the data were concluded independently by two reviewers. Discrepancies were then resolved by a third reviewer. Results are summarised in both chart and narrative form below.

**Results:**

The final 60 publications were then split into three subcategories: (i) GDPR critique (23 publications listed); (ii) iterations of consent and data altruism (21 publications listed); and (iii) proposed solutions and current practices (31 publications). Certain of the publications fell into more than one of the above subcategories, given the interdisciplinary element of the subject and theme of each paper. Throughout the research, 5 of the publications discuss the Data Governance Act and data altruism, with 4 of those providing a critique over the text used in the DGA and the concept of ‘data altruism’ in relation to ‘consent’ as defined within the GDPR and the overall legislative framework for the secondary uses of data.

## Introduction

When the Belmont Commission (1) articulated the necessary conditions for informed consent (2), they might have thought that would be the final word on what informed consent is. Though it is important to note the distinction between ‘consent’ as a theoretical question, ‘consent’ within the context of ethical standards and ‘consent’ as a legal basis for the processing of personal data and their reuse in research, the complexity of the subject, and ambiguous conclusions it has led to, can arguably be seen as common ground.

Over the past few decades, studies and publications show that the collection of data on the basis of precisely informed consent for a single study greatly reduces, and arguably renders impossible, the utility of the data for any future-facing research. The clinical research field had therefore gravitated towards *ad hoc* and, at times, poorly evaluated means of various forms of broadening the consent initially obtained for the collection, processing and use of data, to incorporate the possibility of the downstream reuse of that data (2, 3).

The requirement for researchers, biobanks and clinical trial repositories to obtain informed consent from invited participants in medical research or clinical trials, prior to the beginning of the research, is a fundamental principle of medical research set out in the Declaration of Helsinki (4). However, the Declaration, albeit not legally binding, is only one of the many international instruments drafted and contributed to the debate over ‘consent’ within the field of medical research over the past few decades (5). It is noteworthy emphasize that the requirement of informed consent in a clinical trial context must not be confused with consent as a legal ground for processing personal data, as the former is a safeguard, an ethical standard and procedural obligation in order for a participant to join a clinical trial whilst the later constitutes a legal basis and a means of data processing compliance.

In a data-driven medical research context, before the General Data Protection Regulation (“GDPR”) (6) entered into force, literature shows that participation of data subjects in research was perceived as integrating both ‘interventional consent’ as well as ‘informational consent’ to process personal data (7). Following the introduction of the GDPR, ‘consent’ gained a codified definition with a threshold to be met, as defined within Article 7 and Recital 32 of the Regulation in order for the legal mechanism to act as an exemption to the processing of special category data under Articles 6 and 9.

The practical implementation of informed consent within this context and the high threshold that has to be met in order for an entity or researcher to be able to rely on the legal basis (informed consent having to be clear, concise, specific and granular, freely given and revocable) have been long discussed and highlighted as being a ‘demanding standard’ (8) that has resulted in uncertainty and confusion as to its practical application and how this can be achieved given the very nature of research. An example, which is often quoted and highlights this complexity, is the challenges that meeting this threshold can impose towards biobanks, when in practice the collection of data as a means of resource that can be repeatedly used for research constitutes the foundation of their structure (2). Further issues, such as the failure of informed consent to engage with the individuals involved in the chain of data transfers (9) and the fragmented practices and requirements across the implementation of ‘consent’ at European Member State level, make it difficult for research ethics committees to establish consistent standards as well as for cross-border data transfers and European projects to come to a cohesive understanding and adopt common practices (10, 11).

Though, as also explicitly mentioned in guidance received from the European Data Protection Board (‘EDPB’), consent is only one of six legal bases to process personal data, and the most appropriate choice of a legal basis should be made depending on the circumstances (4, 12), the questions over the practical application of consent for data reuse and processing and how this can be achieved within research remained unaddressed by the legislator. In this light, the introduction of the Data Governance Act (‘DGA’) (13) formally introduces a more relaxed interpretation of consent, by arguably making provisions for a broad consent model, through the introduction of ‘data altruism’.

Data altruism, as defined within the Regulation, is understood to equate to the voluntary sharing of data based on the consent of data subjects to process their personal data, or the permission of data holders, whether natural or legal persons, to allow the use of their non-personal data, without seeking or receiving a reward going beyond any costs incurred to make data available and for objectives of general interest (14). Though very clearly not constituting a legal basis for processing, by introducing the concept of “processing for purposes of general interest,” the DGA may seem to also allow in certain circumstances the processing of personal data for not strictly defined research purposes which serve the general interest. As one of these purposes could include medical and scientific research, it can therefore be observed that the concept addresses the ‘restrictive position of the Article 29 Working Party on broad consent’ (4).

It is within this context that various iterations of consent for personal data processing and reuse have been identified, each varying in description, and addressing the different levels of communication between research participants and researchers, as well as the means of obtaining consent (15). The varying opinions expressed by scholars and academics on these iterations, with advantages and disadvantages of each being measured and preferences shared, can arguably contribute to the conclusion that the questions raised over the practical application of consent, when the GDPR was first introduced, have yet to be answered and that a solution from the legislator is still very much needed.

In the discussion part of the paper the authors, drawing from the DGA and using above mentioned issues between the practical implementation of the lawful basis of ‘consent’ and research, observe and argue that ‘data altruism’ is in fact bringing a significant change to the existing legal framework, going beyond far and few of the national approaches, and holds real promise to create an opportunity to implement consent and the research exemption in a harmonised manner through building a EU-level data altruism mechanism and fostering EU wide trust in data sharing.

## Methodology

In this scoping review, we summarise findings from research conducted in literature, studies and other forms to address and answer the following:

1. What gaps has the GDPR left when it comes to the interpretation and practical application of consent of data subjects towards the secondary use of health data;
2. Can the DGA, through the concept of ‘data altruism’, address these issues and provide a solution;
3. What solutions have been used so far in practice to address this problem.

This rapid evidence synthesis has allowed for the identification of existing practices, models of ‘consent’, as well as a good understanding of the present landscape in terms of data reuse. Further, the research surrounding this review and findings responds to the challenges raised and faced by researchers in this sphere and has provided the authors with an understanding of the role that data altruism can play in this, particularly in light of the upcoming enactment and implementation of the newest addition to the legal landscape for data reuse, the European Health Data Space (EHDS) which will amplify the need for uniform solutions to the present issue in data reuse consistently across Europe.

In order to identify studies relevant to the subject of this review, we have systematically conducted the following research. The search was conducted through the use of six search engines (PubMed, EMBASE, Google Scholar, Web of Science, SAGE and OVID), using a tailored search strategy and through the use of both Medical Subject Headings (MeSH), terms used for indexing, cataloguing, and searching of biomedical and health-related information, and non-MeSH terms and non-MESH terms, as shown in Table 1.

**Table 1:** List of MESH and non-MESH terms.

MESH term	Non-MESH term
Altruism	Data reuse
Informed consent	Secondary use of data
Data sharing	Data donation
Clinical trials	Data altruism
	Broad consent
	Dynamic consent
	E-consent
	Electronic consent
	GDPR
	Data protection regulation
	HIPAA
	Clinical studies
	Consent

Searches were limited to publications from the year 2015 onward in order to capture recent consent-related studies and publications, where the GDPR could act as the main source of legislation for the definition of consent.

The keywords and search queries were run through six search engines and findings were limited to full text publications, which were restricted to those available in English. Results stemming from searches conducted through Google Scholar were limited to the first 15 pages. Websites of EU or international bodies whose work focuses on data sharing and grey literature were also researched [e.g., the European Commission, Committee Reports, Impact Assessments and the Legislative Observatory of the European Parliament, the Towards European Health Data Space (‘TEHDAS’) research project]. No further geographic restrictions were identified.

Results from each database and search engine were imported into Excel and duplicates were removed. The reference lists of included publications or related studies for additional citations were also conducted. Publications related to wellbeing data, IoT data, direct-to-consumer services, social media related, organ donation, posthumous data and consent capacity were excluded from the searches as the themes were out of scope for this paper.

The title-abstract and full text screening and charting the findings were concluded independently by two reviewers and discrepancies were then resolved by a third reviewer. The authors also applied snowball sampling of included studies or related publications for additional eligible materials. The search is presented in Table 2.

**Table 2:** Search strategy.

Search train	PubMed	EMBASE	GS	SAGE	Web of Science (WOS)	OVID	Duplications	Title review	Abstract review
(“reuse of data” OR “secondary use of data”) AND (GDPR OR “data protection regulation”) AND (data altruism OR data donation OR broad consent OR dynamic consent OR e-consent OR electronic consent)	2	3	644	16	2	44	21	47	23
(“reuse of data” OR “secondary use of data”) AND (data altruism OR data donation OR broad consent OR dynamic consent OR e-consent OR electronic consent)	14	19	1,910,0	61	13	266	120	55	45
(“reuse of data” OR “secondary use of data”) AND (GDPR OR “data protection regulation” OR HIPAA) AND (data altruism OR data donation OR broad consent OR dynamic consent OR e-consent OR electronic consent)	2	3	758	20	2	49	77	11	11
(“data sharing” OR “health research” OR clinical studies OR clinical trials) AND (“reuse of data” OR “secondary use of data”) AND (GDPR OR “data protection regulation” OR DGA OR “Data Governance Act” OR HIPAA)	4	9	1,010,0	14	2	114	57	48	32
(“data sharing” OR “health research” OR clinical studies OR clinical trials) AND (“reuse of data” OR “secondary use of data”) AND (data altruism OR data donation OR broad consent OR dynamic consent OR e-consent OR electronic consent)	7	12	1,460,0	39	10	264	138	43	38
**Total**	**29**	**46**	**5,789**	**150**	**29**	**737**	**415**	**361**	**146**

**Figure 1:**
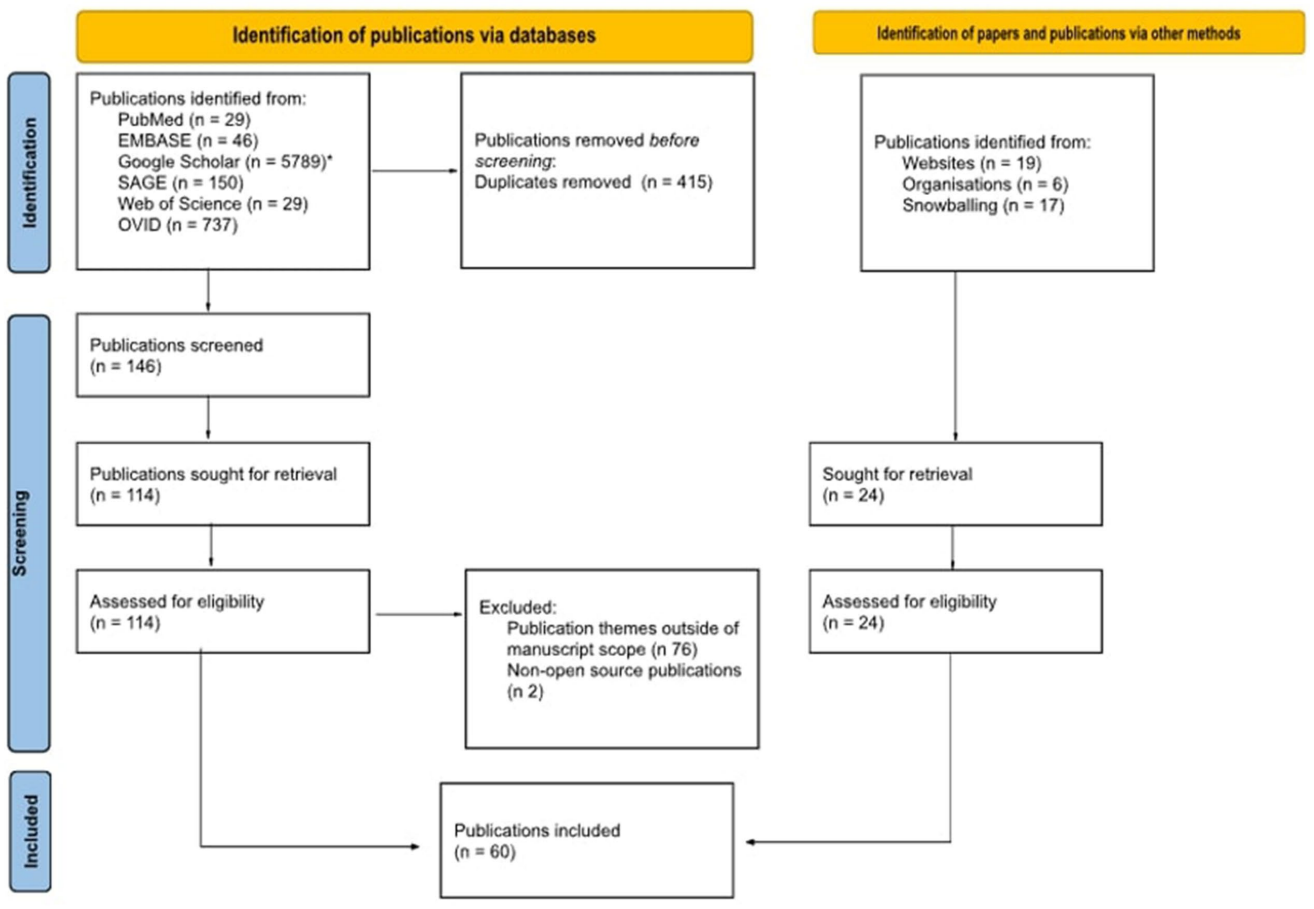
PRISMA chart.

The conducted searches through the use of combined search terms resulted to 6.780 findings, where 415 duplications were identified. The remaining 6.365 publications underwent title review, with 361 publications kept. Subsequently, the remaining findings underwent abstract review which resulted in 146 of those kept and read in full. By excluding publications which were not open access and whose scope was not in line with the parameters of the present research, 36 papers were left. When combined with the publications which were identified via websites, organisations and snowballing, this ultimately resulted to 36 publications remaining as relevant for the purposes of the publication. Figure 1 showcases the process by which the publications were reviewed and the process of the final papers’ inclusion:

The 60 publications were subsequently split into three subcategories—(i) GDPR and ‘consent’ critique as a legal basis, (ii) models of ‘consent’ and data altruism, and (iii) proposed solutions and current practices. Certain publications were listed as part of more than one of the above subcategories, given the interdisciplinary element of the subject.

## Results

### GDPR and the legal basis of “consent” critique

23 of these publications fell under the GDPR critique category, with the majority arguing that the threshold that stakeholders and researchers have to meet to be compliant with informed consent under the GDPR has left many questions and has created obstacles in advancing research.

Part of the findings in this research point towards a fatigue caused by the issues surrounding “consent”, which in turn has given rise to an emerging default practice of “consent or anonymise” (16). This is further supported by the term “consent misconception” (17), where academics create a correlation between consent, as the preferred mechanism used in ethics, equating to consent being the ideal legal basis for processing of personal data. This has been observed specifically in instances where the only alternative to obtaining consent, seen as necessary in specific cases for the reuse of data, given the difficulty in meeting the legal threshold required is the anonymisation of the dataset. Though frequent, this is however not always a suitable, or even correct, approach given that it may render some datasets useless (for example images or medical scans) or may not fit the structure of the entity that conducts the research (such as biobanks and research/trial repositories).

Within this context, the provisions of the GDPR have been argued by some scholars to show support towards a broad consent approach (18), which would favour future-looking research, with participants providing consent to the donation of their data for a ‘type’ of research, without knowing the specific studies to be conducted. Most frequently these arguments on the spirit of the GDPR stem from the wording of Recital 33 supporting that it provides means for a “relaxed approach” to the reading of Article 9 (2)(a) in cases where identifying the exact purpose of research is not evident at the time of data collection. However, the Working Party’s ‘Guidelines on consent under Regulation 2016/679’ (19) as well as the EDPB’s position (20) offer a much stricter reading and scrutiny over the application and interpretation of the relevant articles.

### Models of consent and data altruism

21 publications from the findings discussed and analysed a type of consent, either via comparison to other practices or by arguing for the use of a specific model. The below list showcases the terminology used for the different models identified. The definitions of these models were included in the deliverables of the TEHDAS project (21):

- Informed consent (also referred to as ‘study-specific consent’)
- Implied consent
- Blanket consent
- Broad consent
- Dynamic consent
- Tiered consent
- Meta consent
- Layered consent
- Targeted consent
- Universal consent
- Partnership model of consent
- Opt-it format
- Opt-out format

From the 13 above-mentioned versions of consent, the majority of literature advocate for the use of dynamic or meta consent, with 15 and 6 references, respectively, for each model, in the alternative to the use of broad consent where limitations on this model pose a serious impediment on practical research. Literature preferences for these models as best informed consent practices are expressed on the basis of the continuous engagement of data subjects in the reuse of their data and the level of control that data subjects have over the preferences of how their data is used and shared. In addition to this, both models have been used in connection to electronic informed consent (22), and the use of interfaces and other electronic systems and e-consent tools that support these forms of consent.

The research highlights an interesting juxtaposition between the use of broad consent. With studies quoting up to 98% if its participants being in favour for the utilisation of broad consent in secondary uses of data (23), the model has been described as an imperfect but adequate option for big data resources (24), administratively difficult to achieve and yet still raising questions over the ethical acceptability of less than study-specific consent (25), whilst also seen as satisfying a high ethical standard whilst enabling patients to widely share their data and raise awareness (26, 27).

Further, dynamic consent has also been frequently presented as a preferred option yet criticised as a potential informational burden that participants are asked to carry, especially if not familiar with the IT elements that are frequently engaged (9). Lastly, another version of consent that has been highly quoted in literature is the ‘meta-consent’ model, argued to respect the autonomy of research participants as well as be feasibly achievable for secondary uses of data (28). This model has been argued by scholars to provide as much control as possible to individuals over their own data, yet it has also been heavily criticised for failing to meet the gold standard of consent given that it does not circumvent the unknowability of potential future uses (29) and that, on balance, the costs and practical problems in providing meta-consent to participants outweigh the positive elements.

In addition to the above, throughout the 21 related findings, 5 of the publications discuss the Data Governance Act and data altruism, with 4 of those providing critique over the text used in the DGA and the concept of ‘data altruism’ in light of the ‘consent’ GDPR legal basis and the overall legislative framework for the secondary uses of data. In spite of the fact that the concept of ‘data altruism’ is by no means novel, as related notions such as those of data donation or data solidarity have been long part of the debate on data reuse and sharing for research purposes (30–32), research shows that there is a clear lack of a common understanding or definition in the wider legal and ethical literature. Terms such as “data solidarity” (33), “data donation” (34), “digital philanthropy,” (35) and even “health-information altruists” (36) were part of the findings during the research stage of this publication, and though all seemingly are used interchangeably and speak to the same essence, data being used and benefiting the public, the notions they are based on are in reality rooted in different interpretations. In addition to the definitions found in literature, practical examples of initiatives that resemble the concept of data altruism can be found in national approaches and systems, with the German Patient Data Protection Act and the French Health Data Hub acting as very similar legislation and mechanisms, respectively (27).

### Proposed solutions

Lastly, out of the total, 31 publications discussed and included proposed solutions and best practices, stemming from either research projects or national examples and implementations.

Further to the above findings related to the informed consent practices and models, research results also show that practical solutions currently implemented or proposed by stakeholders and academics can be divided into three categories: (i) endorsement of a practice as the gold standard by the legislator and/or the research community; (ii) creation of task forces and better communication channels between stakeholders and the research community, to benefit and enable from solutions explored in the field of navigating secondary uses of data; and (iii) better engagement of patients and research participants as well as promotion and support of data literacy and means through which the present and proposed solutions in the landscape can benefit them.

Specifically, academics suggest the creation of a GDPR-compliant broad consent standard (37) which, complemented by national, international and EU practices, gives rise to harmonised models of consent forms and safeguards enabling cross-border sharing and transfer. In addition, the creation of ‘participation pacts’, ‘social contracts’ and consortia or tasks forces between researchers and donors (38–40) have been raised as potential solutions for the past decade as an attempt to provide harmonisation over the requirements as well as clarity over the reasonable expectations for secondary health data reuse.

In their pursuit for a solution which would accommodate the research community as a whole, the findings show that researchers have developed a variety of electronic tools and models to facilitate the capture of consent in a way to accommodate as many of the iterations as possible. Though the practical use of eIC (‘electronic informed consent’) is still relatively uncommon (41), it has been documented (16) as having the potential to not only harmonise the utilisation of a single tool at an EU-level but has been supported as means of establishing a clear and constant communication channel between research participants and researchers. To add to this, guidance documents setting out the requirements and limitations to the use of eIC, which is increasing in the last four years, in specific cases have been developed, though these practices are observed to so far be adopted, these vary between institutions (16) and the adoption of a harmonised approach across stakeholders can be challenging when considering the reluctance expressed by some Member States for the use of eIC in clinical trials (42).

Drawn from the findings above, the authors conclude that, regarding the questions which have formulated the research conducted, the gaps left behind in the practical application of ‘consent’ as a legal basis under the Art. 6 and 9 of the GDPR are still very much present and constitute an obstacle that the research community is trying to address in a variety of means, without a clear winning answer endorsed. The Data Governance Act, through the concept of ‘data altruism’, could indicate a solution through which the scientific research exemption and consent as legal basis are applied into practice. However, its application, interplay with the GDPR and a common understanding of the concept in the wider clinical, legal and ethical literature are questions that still remain unanswered. Insofar as the solutions proposed and found in literature, the authors endorse the notion of the proposed preferred models and support the need for a unified approach. A discussion and further analysis of the conclusions drawn by the authors and findings can be found below.

### Discussion and legal analysis

As highlighted in the research findings, the obstacles in obtaining consent and meeting the necessary legislative threshold for the secondary uses of personal data have been extensively discussed in literature (19). The actual possibility of “meaningful” and compliant consent being obtained at the data collection stage, with both participant and research having full access to the information and understanding of what consenting implies, and the practical realities of how this looks from a costs and time–pressure perspectives have been highlighted both in publications (43) as well as in discussions taking place between stakeholders (44).

The identification of the 13 consent models which are attributed to one GDPR legal basis, and the respective longstanding debates between scholars on the positive and negative attributes that each carries, echo the fragmentation that exists in the research community’s question which has left unanswered since the first draft of the GDPR text. Though the GDPR has provided legal certainty over data protection aspects, it has yet to resolve or sufficiently pragmatically address the key problem of informed consent. In addition, part of the reasoning as to why these models have yet to be translated into widespread practice is due to the fact that it is difficult to envision how they would play with other national restrictions in addition to the European legislative landscape and other practices used in the field. As a response to these issues, the variety of adapted models of informed consent have been developed and identified to have come on the rise as a ‘shift’ from specific consent and with aim to cover a range of future data uses and level of involvement of data subjects.

Given the uncertainties, both on a legal and ethical front, raised by the wide use of broad consent many publications argue in favour of dynamic consent, as an approach that arguably meets “the gold standard” (45), being consistent with the legal requirements required for participants to opt-in for each new reuse of their data in a study, and promoting the active role of the data subject. However, complications may arise in widely adopting this approach taking into consideration the recently agreed European Commission proposal for a European Health Data Space (EHDS) (46), which will equate to a big part of this landscape having to undergo important changes. In the same light, the scalability of dynamic consent can arguably also be questioned given that in practice, individuals engaged in projects will receive numerous requests requiring a positive/negative response yet somehow having to achieve the informed consent threshold.

Though the EDPB’s clarification (39) that ‘consent’ is not the only legal basis that can be relied on for those engaging in scientific research has been welcomed by stakeholders (47), the authors argue that this by no means provides answers to the questions or obstacles faced around the subject particularly when considering that in practice the broad consent model could enable innovation and future looking research whilst balancing both patients’ rights and level of engagement. Other legal bases such as the processing of data necessary for the public interest or for scientific research (Articles 9 (2)(g) and (j) respectively) have been highlighted as carrying additional practical difficulties when analysed through the lens of cross-border sharing and research. Where data processors have to consider the legal specificities of two or more Member States, additional requirements and diverging rules in national approaches, for example for the use and reuse of genomic data in research, have been described as contributing to barriers and obstacles in a fragmented cross-border legislative tapestry (18). An example of this is the diverging approach taken by Latvia and Germany and Latvia, where the former has implemented national specific legislation governing genomic research whilst the latter does not (48). Interestingly, the European Parliament study conducted in 2023 (49) highlighted some of these issues. However, in spite of the proposed solutions and efforts, no permanent solution has been found or implemented. The authors believe that these additional requirements stem from Article 9(4) GDPR, though providing flexibility to Member States to define their own rules for data sharing and enabling national approaches to be integrated into the regulatory framework following the adoption of the GDPR, do indeed contribute to the difficulties faced by researchers and stakeholders in this field.

In this light, the introduction of the DGA could arguably be a light at the end of the tunnel offered by the legislator in the form of a more relaxed approach to the interpretation and application of consent. Through the introduction of data altruism, the DGA is bringing a significant change to the existing legal framework and holds real promise to create an opportunity to implement the legal basis of consent as well as the research exemption in a harmonised manner through building a EU-level data altruism mechanism and fostering EU wide trust in data sharing. Despite existing legal frameworks found in Member States resembling the concept, data altruism is set apart by having the ability to build an EU-wide data sharing altruism mechanism that holds clear promise toward harmonisation. Whether the interpretation of ‘consent’ leans towards a broad consent or not, broad consent being understood as a model of consent which permits for the collection, processing and reuse of data for research that cannot be specified in detail due to its nature, through the introduction of data altruism the DGA may also enable in certain circumstances the processing of personal data for not strictly defined research purposes which serve the general interest, and ultimately enable future looking research. Whilst very clearly not constituting a legal basis for processing, it can also be observed that data altruism could address the ‘restrictive position of the Article 29 Working Party on broad consent’ and create an opportunity to implement the research exemption at an EU level, as one of these purposes could include medical and scientific research (4).

Related to the foregoing, informed consent forms have long been criticised due to their long, jargon-filled format, argued to be frequently used as mere means of obtaining consent and undermining the original aim of being understood by research participants (50, 51). Yet, through the introduction of the European data altruism consent form, under Article 25 of the Regulation, individuals and entities engaged in data altruism and data sharing will be able to have a tangible point of reference as to the requirements needed for the collection of data across Member States in a uniform format, including the informed consent model endorsed by the EDPB and European Data Protection Supervisor (‘EDPS’), members sitting at the European Data Innovation Board. Though the subject was raised during a meeting of the EDIB in early 2024, with a noteworthy mention that researchers would find appealing the broad consent model to be deployed, no further materials are still expected to be shared (52).

In any event, for the successful use of eIC and eConsent tools to be deployed widely and successfully, the ICT tool developed to support in the capture of consent for whichever model chosen would be required. For this to work, an interactive interface that, contributing to the empowerment of the participant in being in control of their data, would allow them to choose and alter their consent choices in real time whilst simultaneously also conforming to the compliance required in both its design and use. Projects such as IDERHA (53), EnCore, HELEX, REg4ALL (54), using broad and dynamic consent as paradigms, have developed a proposed tool which tailors consent options to adhere to the legal requirements whilst ensuring, through keeping the participants in the flow of data, data sharing is enabled. In conjunction to this, a consent matrix (47) has also been proposed as an efficient method for collecting metadata on conditions surrounding consent which, when compared with best practices in the development of consent forms. The use of an eIC alongside the data altruism consent form, as contained within Article 25(4) DGA, and the model of consent used by the legislator in the particular form could act not just as paradigm but indeed as the way forward to tackling the issues of obtaining and reuse data on the basis of consent. The provisions of the DGA referring to the creation of a rulebook through a delegated act, though indeed not a hard requirement in regards to uniform application (55), could also address and bring to light the objectives that the community wishes to achieve through the creation of societal compacts and participation packs. To contribute to the changes in the present legal framework, through the implementation of the DGA across Member States, monitoring bodies tasked with ensuring compliance to the requirements of the Regulation can ensure harmonisation of the data altruism mechanism is achieved through the application of data altruism alongside national requirements and by uniting the efforts made currently on a national or consortium level and bringing them to an EU-wide stage.

The EHDS aims to enable an efficient exchange of and direct access to different health data across the Union in compliance with data protection regulations, in particular the GDPR (43, 56, 57). The EHDS Regulation, though not adopted or implemented yet (58), has placed strong emphasis on stepping away from the use of ‘consent’ as the legal basis for the secondary use of data and instead (59), through the text of Recital 37, aligns the legal basis with Articles 9(2) (g), (h), (i), and (j) GDPR. It could arguably be implied that through this action, the legislator acknowledges the practical difficulties in the secondary uses of health data and the fact that a concrete solution to the issue is very much still present.

With this in mind it is important to note that the EHDS Act makes space for an opt-out mechanism, allowing patients and citizens to actively object to the secondary use of their data. Though this has been overall welcomed by stakeholders (60), this mechanism is left at Member States’ discretion which arguably would be counterintuitive and go against the harmonisation of the application and interpretation of the overall Proposal. Further, though the EHDS by no means alters the legal status and interpretation of consent, its addition to the data reuse landscape will undeniably augment both the demand as well as the opportunity for the reuse of data. Consequently however, it will also increase and highlight the need to provide solutions to the issues faced through the use of ‘consent’ as a legal basis for secondary data uses in the event where, as part of the EHDS implementation, the particular basis is used by Health Data Access bodies in relation to datasets they made accessible. Within this context, it is therefore paramount to ensure that data altruism is a mechanism that benefits the community in sharing health data and is seen as not an additional legal basis circumventing the existing GDPR requirements but rather as means of obtaining consent with qualifiers such as the minimisation of risk of informational harm, offering ethical protection and enabling researchers to conduct forward looking research whilst empowering individuals by having control over their data.

### Strengths and limitations

The present publication, to the best of the authors’ knowledge, serves as the first cohesive and conclusive research of the data altruism mechanism and the interconnections with the lawful basis of consent under the GDPR in health data reuse. However, several limitations could be listed for this type of study, given the topic’s close connection to data reuse and the EHDS’s implementation will likely give rise in many more search results with the implementation of the DGA and inputs from the legislator over time.

The DGA framework and data altruism concept are aimed at a variety of sectors and not exclusively towards health data reuse of clinical research, making by extension the findings and conclusions of the present paper perhaps not directly applicable in another context. In this regard, the searches conducted purposely excluded publications related to wellbeing data, direct to consumer services and products as well as consent capacity of individuals and social media related data protection and data uses.

It is important to further highlight that the consensus identified through the uses of the identified models (dynamic, meta or broad consent) does not equate to unanimity. Further qualitative research is required to identify the operational basis and practical implementation of data altruism and the selected model in the field of secondary uses of health data as applied and viewed by stakeholders, data altruism organisations or health data access bodies. Research in relation to the specific consent model to be chosen and deployed eventually by the EU legislator at the stage of implementation of the DGA would also be useful. In addition to this, an important limitation is the discussion on the EHDS, as the Proposal is yet to be neither adopted nor implemented on an EU or national level.

## Conclusion

What is paramount to note is that addressing issues related to secondary uses of data in research cannot be solved by *ad hoc* efforts, whether that be administrative, legal, or policy changes. Rather multidisciplinary discussions in line with the international character of high-level research need to take place (61), involving stakeholders, patients, researchers and the legislator. In this light, we note that the shortcomings of the abovementioned suggested solutions and benefits of such approaches could be aided to come fruition and maximised when paired with the data altruism framework. Support for digital literacy is vital for solutions to the pending issue to be successful in a wide EU setting in order to promote public trust and ensure the transparent and efficient use of electronic platforms and relevant tools (17), one could argue that through the data altruism framework and the text of Article 20, this could be achievable.

Though at present it is as easy to speculate that data altruism consent is another drop of water in the ocean (62), as easily as it is to argue that the concept is a solution long-waited for, the DGA itself and its application are both still at their infancy. Questions on the interplay between data altruism, the GDPR requirements, and the practical implementation of GDPR’s consent as an overall requirement for participation in clinical trial research remain at best unclear and vague (63). What cannot be disputed is that the DGA and the altruism mechanism carry promise and have already been labelled, in studies and literature, as a significant change in the practical landscape of health data research (26, 64).

The detailed review of existing literature and practices contained in this paper may be used as the basis to expand on this area and identify priority areas for further exploration in a subsequent empirical research. The questions have been drafted on the basis of an initial review of the literature and feedback from subject matter experts. Findings could also be used to develop a research agenda for better addressing of key concerns in the application of data altruism and broad consent for future use, including data subject engagement, transparency, and uniform applicability and benefit sharing.
